# Incidence and predictors of Lhermitte’s sign among patients receiving mediastinal radiation for lymphoma

**DOI:** 10.1186/s13014-015-0504-7

**Published:** 2015-09-25

**Authors:** Bassem Youssef, JoAnn Shank, Jay P. Reddy, Chelsea C. Pinnix, George Farha, Mani Akhtari, Pamela K. Allen, Michelle A. Fanale, John A. Garcia, Patricia H. Horace, Sarah Milgrom, Grace Li Smith, Yago Nieto, Isadora Arzu, He Wang, Nathan Fowler, Maria Alma Rodriguez, Bouthaina Dabaja

**Affiliations:** Departments of Radiation Oncology, The University of Texas MD Anderson Cancer Center, 1515 Holcombe Blvd, Houston, TX 77030 USA; Departments of Lymphoma & Myeloma, The University of Texas MD Anderson Cancer Center, Houston, TX USA; Departments of Stem Cell Transplantation, The University of Texas MD Anderson Cancer Center, Houston, TX USA; Department of Radiation Oncology, The University of Texas Medical Branch at Galveston, Galveston, TX USA

## Abstract

**Purpose:**

To prospectively examine the risk of developing Lhermitte’s sign (LS) in patients with lymphoma treated with modern-era chemotherapy followed by consolidation intensity-modulated radiation therapy.

**Methods:**

We prospectively interviewed all patients with lymphoma who received irradiation to the mediastinum from July 2011 through April 2014. We extracted patient, disease, and treatment-related variables from the medical records of those patients and dosimetric variables from treatment-planning systems and analyzed these factors to identify potential predictors of LS with Pearson chi-square tests.

**Results:**

During the study period 106 patients received mediastinal radiation for lymphoma, and 31 (29 %) developed LS. No correlations were found between LS and any of the variables examined, including total radiation dose, maximum point dose to the spinal cord, volume receiving 105 % of the dose, and volumes receiving 5 or 15 Gy.

**Conclusion:**

In this group of patients, treatment with chemotherapy followed by intensity-modulated radiation therapy led to 29 % developing LS; this symptom was independent of radiation dose and seemed to be an idiosyncratic reaction. This relatively high incidence could have resulted from prospective use of a structured interview.

## Background

Radiation therapy (RT) to the mediastinum and thoracic regions, especially to the heart and lungs, has been associated with various forms of toxicity [1–4]. Another less well studied symptom, Lhermitte’s sign (LS), has been linked with RT to the spine cord. LS, known colloquially as “barber’s chair phenomenon,” manifests as a transient electric shock-like sensation or tingling in the neck that radiates down the spine and into the extremities. It can occur spontaneously or can be triggered by movements such as neck flexion, walking, or, less commonly, by extension or rotation of the neck [5, 6]. LS was first observed in patients with multiple sclerosis by Marie and Chatelin in 1917 but was not recognized by the neurology community until it was reported by Jean Lhermitte in 1924 [7]. Although LS is most commonly associated with multiple sclerosis, it can also occur in other demyelinating conditions such as neuromyelitis optica or as a side effect of RT to the cervical or thoracic spinal cord [8–10]. The latter condition, termed radiogenic LS, is caused by reversible demyelination of ascending sensory neurons at the dorsum columns due to inhibition of oligodendrocyte proliferation after irradiation of the cervical or thoracic spinal cord [11–13]. Once the oligodendrocytes recover and myelin is resynthesized, the symptoms subside. The symptoms of LS usually begin within a few months of completion of RT and are transient. This is in stark contrast to radiation myelitis, in which symptoms generally develop 1 year or more after radiation and progress to permanent spinal cord injury. Although LS is not usually associated with chronic progressive irreversible myelitis, delayed radiation myelopathy causing paralysis may be preceded by LS [14].

The reported incidence of LS among patients receiving two-dimensional (2D) RT without chemotherapy to the cervical or thoracic spinal cord has ranged from 3.6 to 13 % [10, 14–16]. However, a recent study reported LS in 21 % of patients receiving chemotherapy concomitant with intensity-modulated radiation therapy (IMRT) for head and neck tumors [17]. Reasoning that patients receiving RT for lymphoma may also be at increased risk of LS, we prospectively examined the incidence of LS in a series of consecutive patients treated with RT to the mediastinum and thorax, and sought to identify potential predictive factors for the occurrence of radiogenic LS.

## Methods

After receiving institutional review board approval to conduct this study, we prospectively identified and followed 116 consecutive patients with a confirmed diagnosis of lymphoma treated with chemotherapy followed by consolidative RT to the mediastinum between July 2011 and April 2014 at a single institution. Ten patients had to be excluded since we could not get in touch with them and they failed to come back for a follow up. All patients but one were treated with IMRT using a “butterfly” field arrangement to sites involved with disease [18]. We reviewed the type and number of chemotherapy cycles. All patients were given structured interviews at the completion of RT and at every follow-up visit, either in person in the clinic or by phone for those who did not return for follow-up. The timing for follow up was every 3 months for 2 years for those who did not develop the sign, and until resolution of symptoms for those who did. Specific questions were asked about the development of LS as follows: “have you experienced any shooting-like pain, feeling of electric-like shocks or sharp pain in your neck or back that radiates to your arms or legs,” patients who answered yes were further asked about the date of occurrence, intensity, and duration of symptoms. Other questions were asked about the presence of neuropathy, tingling, in their hands and feet as well as symptoms of bleomycin toxicity (in patients who received bleomycin), such as shortness of breath, coughing, and or low-grade fever. These are the symptoms linked to the use of Bleomycin, an antibiotic agent with antitumor activity, including bronchiolitis obliterans with organizing pneumonia, esosinophilic hypersensitivity. Patient characteristics extracted from the medical records included age, sex, histopathologic diagnosis, site of disease, type of chemotherapy, and RT modality used. Other dosimetric information retrieved from RT treatment-planning systems included total radiation dose, dose per fraction, and spinal cord volumes treated to the full prescription dose (V_100%_) and 105 % of the prescribed dose (V_105%_), to 5 Gy and 15 Gy (V_5_ and V_15_), and the maximum point dose (D_max_). Neuropathy and bleomycin pneumonitis, both considered dose independent were evaluated, this was done in an effort to record other toxicities related to therapy. For instance, neuropathy potentially related to vinblastine, hypothesizing that side effects such as these are similar to LS in their unpredictability and lack of dose dependence. Clinical and dosimetric factors were compared by using Fisher exact test for patients who did or did not develop LS.

## Results

### Patient characteristics

Patient characteristics are shown in Table 1. During the study period, a total of 106 patients met the stated inclusion criteria and were prospectively followed. The median age at diagnosis of primary disease was 34 years (range 18–61 years). Most patients (57 %) were female; and 82 had Hodgkin lymphoma and 24 non-Hodgkin lymphoma. Of the 24 non-Hodgkin lymphomas, 11 were diffuse large B cell, 4 were T-cell lymphoblastic, 5 primary mediastinal, 1 gray zone, 1 marginal zone, and 2 mixed follicular and large B-cell lymphomas. Sixty-eight patients received ABVD chemotherapy (doxorubicin, bleomycin, vinblastine, and dacarbazine), of whom 27 patients did not receive bleomycin. Other chemotherapy regimens included rituximab, cyclophosphamide, doxorubicin, vincristine, prednisone [R-CHOP]; hyperfractionated cyclophosphamide, vincristine, doxorubicin and dexamethasone [R-HCVAD], or R-EPOCH by adding etoposide. The risk of LS did not seem to be associated with type of chemotherapy or number of cycles (*p* = 0.43), Table 2 details the chemotherapy regimens and number of cycles used.

**Table 1:** Patient characteristics

Characteristic		Value or no. of patients (%)	
		No LS (*n* = 75)	With LS^a^ (*n* = 31)
Age, years			
	Median (range)	37 (19–73)	32.0 (18–61)
Sex			
	Female	40 (53)	21 (68)
	Male	35 (47)	10 (32)
Ethnicity			
	Caucasian	59 (78.6)	25 (81)
	African-American	4 (5.4)	2 (6)
	Hispanic	10 (13.4)	4 (12)
	Mid-eastern	2 (2.6)	0
Histology			
	Hodgkin lymphoma	56 (75)	26 (84)
	Non-Hodgkin lymphoma	19 (25)	5 (16)
Disease stage			
	I	3 (4)	3 (10)
	II	55 (73.5)	24 (77)
	III	2 (3)	2 (6.5)
	IV	4 (5)	2 (6.5)
	Recurrent	3 (4)	
	Refractory	6 (8)	
	Unknown	2 (3)	
Radiation technique			
	IMRT	71 (95)	29 (94)
	IMRT & 3D AP/PA	3 (4)	1 (3)
	Protons	1 (1)	1 (3)
RT dose, Gy			
	Mean (range)	32.9 (20–46)	33.2 (30.6–46.6)
	≤30.6	49 (65)	22 (71)
	>30.6	26 (35)	9 (29)
	30–36	10 (13)	2 (6.5)
	36–45	16 (22)	7 (22.5)
Peripheral neuropathy^b^			
	Yes	35 (47)	16 (52)
	No	39 (52)	14 (45)
	Unknown	1 (1)	1 (3)
Bleomycin toxicity^b^			
	Yes	62 (83)	7 (23)
	No	3 (4)	24 (77)
Decreased lung function^c^			
	Yes	10 (13)	11 (35)
	No	65 (87)	20 (65)

*Abbreviations*: IMRT, intensity-modulated radiation therapy; 3D AP/PA, three-dimensional conformal radiation therapy with anteroposterior/posteroanterior fields

^a^LS at some time during follow-up

^b^After chemotherapy but before radiation therapy

^c^Out of the 106 patients pulmonary function tests were performed in only 43 patients

**Table 2:** Details of the chemotherapy regimens and number of cycles between patients with and without LS

Chemotherapy	No LS	LS
2–4 ABVD/AVD	24	9
>4 ABVD/AVD	22	11
≥6 R-CHOP	2	1
≥6 R-EPOCH	6	2
≥6 HyperCVAD	1	0
Salvage	20	7

ABVD/AVD: Doxorubicin, Bleomycin, Vinblastine, Dacarbazine

R-CHOP: Rituximab, Cyclophosphamide, Doxorubicin, Vincristine, Prednisone

R-EPOCH: Rituximab, Etoposide, Prednisolone, Vincristin, Cyclophosphamide, Doxorubicin

HyperCVAD: Cyclophosphamide, Vincristine, Doxorubicin, Dexamethasone, Methotrexate, Cytarabine

Salvage: Multiple lines of chemotherapy +/− Stem cell transplantation

**Fig. 1:**
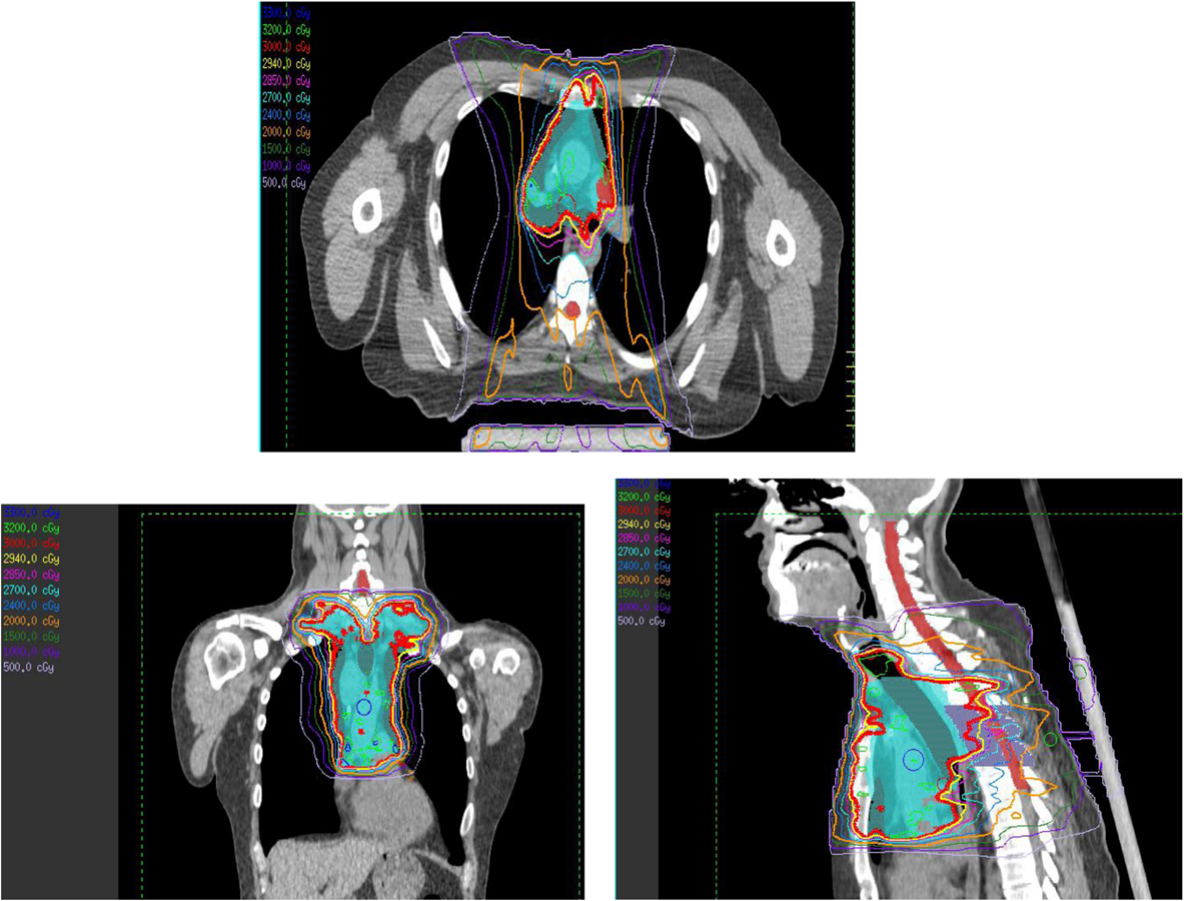
Representative axial (top), coronal (left), and sagittal (right) views of treatment plans for a patient receiving intensity-modulated radiation therapy for mediastinal lymphoma

All patients but one received radiation to involved sites only, the current accepted standard of care, and all but two received IMRT (Fig. 1), [19, 20]. Additional techniques such as use of an inclined board [21], inspiration breath-hold, and butterfly IMRT planning [18] were used to spare dose-limiting structures within the radiation field including heart, lung, and breasts.

### Incidence and risk factors for Lhermitte’s sign

Thirty-one patients (29 %) developed LS. Of those patients, 22 received 30.6 Gy in 17 fractions, one received 36 Gy in 20 fractions, and one 39.6 Gy in 22 fractions; the other 7 patients were treated with a sequential boost technique, with total doses ranging from 36 Gy to 46.6 Gy. The mean prescribed dose was 33.2 Gy (range 30.6 − 46.6 Gy), and the spinal cord D_max_ was 33.5 Gy (range 28.0–43.5 Gy). Nine patients had measurable “hot spots” (defined as ≥105 % of the prescribed dose) in the spinal cord, with a mean volume of 2.7 cm^3^ (range 0.02–14.6 cm^3^) (Table 3).

**Table 3:** Spinal cord doses in 31 patients who developed Lhermitte’s sign

	Total dose,	Spinal cord	Location of	
Patient ID	Gy	D_max_ Gy (%)	D_max_	Cord V_105%_, cm^3^
1	30.6	33.04 (108)	T4–T5	3
2	30.6	28.51 (93)	—	0
3	30.6	33.98 (111)	T3	2.26
4	30.6	29.1 (95)	—	0
5	30.6	31.84 (104)	T5–T6	0
6	30.6	35.81 (117)	T3–T8	14.6
7	30.6	31.02 (101)	—	0
8	30.6	30.10 (98)	—	0
9	30.6	29.93 (98)	—	0
10	30.6	34.66 (113)	T5	0.5
11	30.6	30.85 (101)	—	0
12	30.6	31.96 (104)	—	0
13	30.6	28.06 (92)	—	0
14	30.6	30.94 (101)	—	0
15	30.6	34.67 (113)	T4–T7	1.1
16	30.6	30.01 (98)	—	0
17	30.6	32.18 (105)	T9	0
18	29.75	31.19 (105)	—	0
19	30.6	31.72 (104)	—	0
20	30.6	33.12 (108)	T4	0.8
21	30.6	33.06 (108)	T4	1.3
22	30.6	37.23 (105)	—	0
23	30.6	38.78 (108)	T6	0.02
24	30.6	36.21 (101)	—	0
25	37.4	39.34 (105)	—	0
26	39.6	34.13 (109)	T1-T2; T5; T8	0.8
27	41.4	38.69 (93)	—	0
28	42.0	31.02 (74)	—	0
29	46.6	39.40 (85)	—	0
30	41.4	43.51 (105)	—	0
31	36.0	35.48 (99)	—	0

D max = maximum point dose

**Fig. 2:**
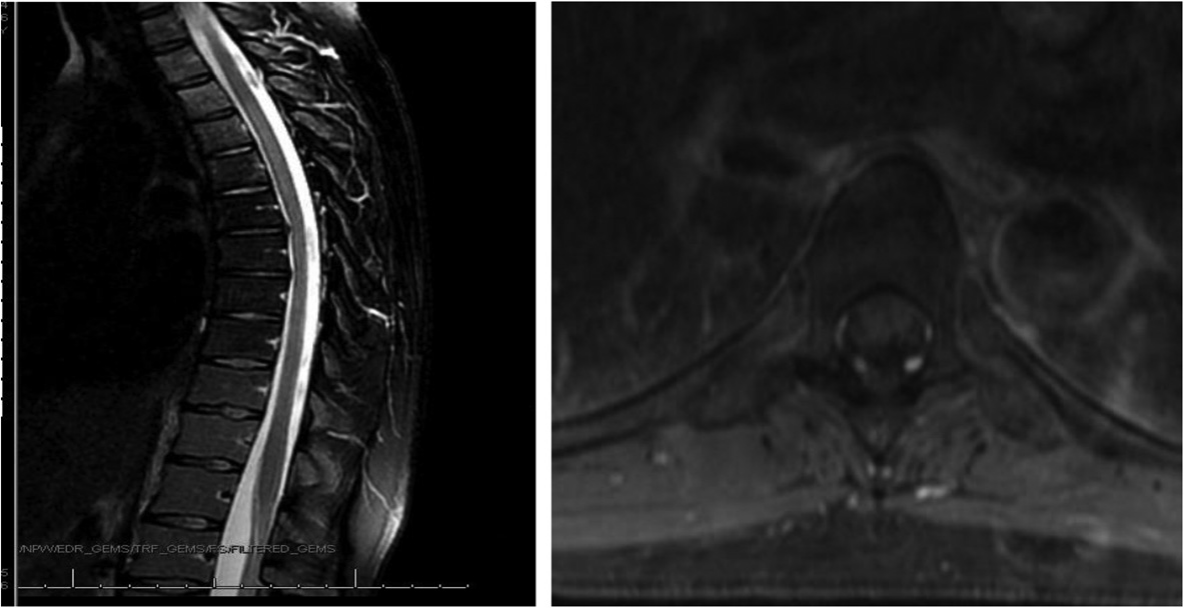
T1-weighted sagittal (left) and axial (right) magnetic resonance images with contrast show dilated subdural veins in a patient with Lhermitte sign and severe symptoms upon walking

The mean time to development of symptoms was 3 months (range 2 weeks to 5 months) after completion of RT; however, one patient developed LS after completing chemotherapy and before starting RT. The average time to noticeable improvement was 8 months (range 2–16 months), but 5 patients had ongoing symptoms for up to 1 year after finishing RT. One patient with severe symptoms evoked by walking for short distances was assessed by magnetic resonance imaging (MRI) of the spine twice, at the onset of symptoms and 6 months later; both MR images showed prominent dilated veins but no sign of demyelination (Fig. 2).

At the completion of chemotherapy but before RT was begun, 16 of 31 patients with LS (52 %) and 35 of those without LS (47 %) had peripheral neuropathy; 11 of 31 patients (35 %) with LS had pulmonary function decreased relative to baseline at that time, which in 7 patients was thought to reflect bleomycin-induced lung toxicity (bleomycin was subsequently discontinued). Because pulmonary function tests were completed by only 43 of the 106 patients at both time points (and for those who received ABVD, at the discretion of the treating physician), we could not compare pulmonary function between groups that did or did not develop LS.

Finally, we evaluated several dosimetric variables, including the spinal cord volume (cm^3^) that received 5 Gy, 15 Gy, or 105 % of the prescribed dose as well as the maximum point dose, for their potential association with the development of LS (Table 4). None of these variables showed any statistically significant relationship with LS.

**Table 4:** Comparison of dosimetric variables and treatment-related toxicities between patients with and without LS

				*P*	*Test*
	All Patients	No	LS	Value	
Characteristic	(*n* = 106)	LS (*n* = 75)	(*n* = 31)		
Volume receiving 5 Gy, cm^3^					
Median	29.9	29	31	0.25	Median
Mean	32.8	33.2	31.7		
Range	9.7–233	9.7–233	15.3–56.5		
Volume receiving 15 Gy, cm^3^					
Median	23	22.6	25.9	0.25	Median
Mean	24.8	24.5	25.4		
Range	8.8–51.7	8.8–51.7	13.2–43		
Spinal cord maximum dose, Gy					
Median	32.2	32.2	33	0.97	Median
Mean	32.7	32.4	33.5		
Range	18.2–47.2	18.2–47.2	28–43.5		
Spinal cord maximum dose, %					
Median	100	100	100	0.33	Median
Mean	99	98	100		
Range	64–118	64–118	74–117		
Bleomycin toxicity					
No	62	45	17	0.40	Fisher’s exact
Yes	17	10	7	0.37	
No bleomycin	27	20	7		
Peripheral neuropathy					
No	53	39	14	0.43	Fisher’s exact
Yes	53	36	17	0.51	

## Discussion

In this prospective evaluation of LS after modern-day RT for patients with lymphoma, we found an incidence of 29 % with a median time to development of 3 months, but we could not identify any baseline or dosimetric characteristics associated with its development. At 29 % this rate was higher than those of previous studies involving use of 2D RT (3.6–13 %) or chemoradiation with IMRT for head and neck cancer (21 %) [17], despite our use of relatively low radiation doses.

The lack of correlation between radiation dose or fraction size and LS in our study is in contrast to previous studies in which LS was linked with higher dose per fraction (≥2 Gy/fraction), spinal cord doses >45 Gy, and altered fractionation [15]. One group, for example, found that a V_45_ of the cervical spinal cord of ≥14.15 cm^3^ was associated with LS in patients with laryngeal or oropharyngeal cancer [22]. Another group showed that fractions larger than 2 Gy were also associated with LS [16]. The lack of correlation in our study, in which 1.8-Gy daily fractions were used with a spinal cord D_max_ of 43.5 Gy, suggests that additional factors contribute to the development of LS, or that LS is idiosyncratic. Also, 11 of 31 of patients who developed LS also developed decreased lung function attributed to bleomycin, which has been established by others as an allergic pulmonary reaction [23–25]. It is worth to add here that we chose to evaluate cord volumes instead of percentages because the entirety of the cord is not usually included in the treatment plans, and thus the volume of the cord receiving a certain dose may be more accurate than the percentage of the cord, which is generally provided in the dose-volume histogram.

Our study also differs from others in that previous reports of LS focused on patients with cancer of the head and neck, whereas our study was limited to patients who received radiation to the thorax and thoracic spine [17]. Also, concurrent chemoradiation has been linked with higher LS incidence, perhaps due to disruption of the blood–brain barrier by radiation and subsequent penetration of cytotoxic agents to the central nervous system [26]. In our study, all patients received systemic chemotherapy, but the chemotherapy was delivered before the RT, so the aforementioned rationale does not seem apply in this case. Third, some case reports suggest that chemotherapy alone, especially cisplatin and docetaxel, given alone or after stem cell transplantation, can cause LS [27–30]. However, use of ABVD has not been associated with LS, and thus the contribution of this type of chemotherapy, if any, to the development of LS in patients with lymphoma remains unclear. Increasing age has been linked inversely with LS, with one study showing younger age to be a risk factor for developing LS [22] and another showing a LS to be less common among patients >60 years old [10]. The young age of the patients in the current study (median 32 years in the group with LS and 37 years in those without LS) may explain the relatively high incidence of LS in this study.

One caveat in interpreting our results is bias introduced by our prospective use of interviews at each follow-up visit, which may have increased patients’ awareness of subclinical LS or related symptoms. Indeed, several patients made comments such as “so that’s what that was.” Another shortcoming of our study was our inability to validate previously identified risk factors for LS development, leading us to speculate that LS is not dose-dependent, as is true for neuropathy secondary to vincristine or vinblastine, or declines in lung function attributable to bleomycin. Nevertheless, this is one of the few studies to examine the incidence of LS after chemotherapy and radiation for lymphoma, and it is the only study to our knowledge to be conducted prospectively.

## Conclusions

This report represents the first prospective evaluation of LS after consolidation RT for patients with lymphoma. We observed a 29 % incidence of LS with a median time to development of 3 months. However, we could not identify any baseline or dosimetric characteristics associated with its development, leading us to conclude the development of LS in this clinical scenario may be an idiosyncratic reaction independent of radiation dose or fraction size. Our findings add to the body of the literature on the occurrence of LS, and confirmation of our findings by others would be helpful for determining the significance of LS among patients receiving thoracic radiation.

## Consent

Written informed consent was obtained from the patient for the publication of this report and any accompanying images.
